# Quantifying the Over-retraction (Ore) phenotype in *C. elegans* male tail tip morphogenesis

**DOI:** 10.17912/micropub.biology.000830

**Published:** 2023-05-01

**Authors:** Julia Burnett, E. Jane Albert Hubbard

**Affiliations:** 1 Department of Cell Biology, NYU Grossman School of Medicine

## Abstract

Morphometrics, the quantitative analysis of biological structures, reduces subjectivity and increases reproducibility in characterizing morphological phenotypes. In *C. elegans* males, the rounded adult tail tip emerges from a stage-specific retraction of epidermal cells regulated by the heterochronic pathway via LIN-41/TRIM71. Precocious tail tip morphogenesis in *lin-41* reduction-of-function conditions results in a blunted tail (Ore) phenotype, previously described qualitatively (Del Rio-Albrechtsen et al., 2006). We present a quantitative method to assess the Ore phenotype by measuring the tail tip position relative to the cloacal opening. This method can be used to study variation in Ore phenotypes and to validate *lin-41* loss-of-function reagents.

**Figure 1. Retraction Angle distinguishes Ore from non-Ore male tail tip phenotypes:**
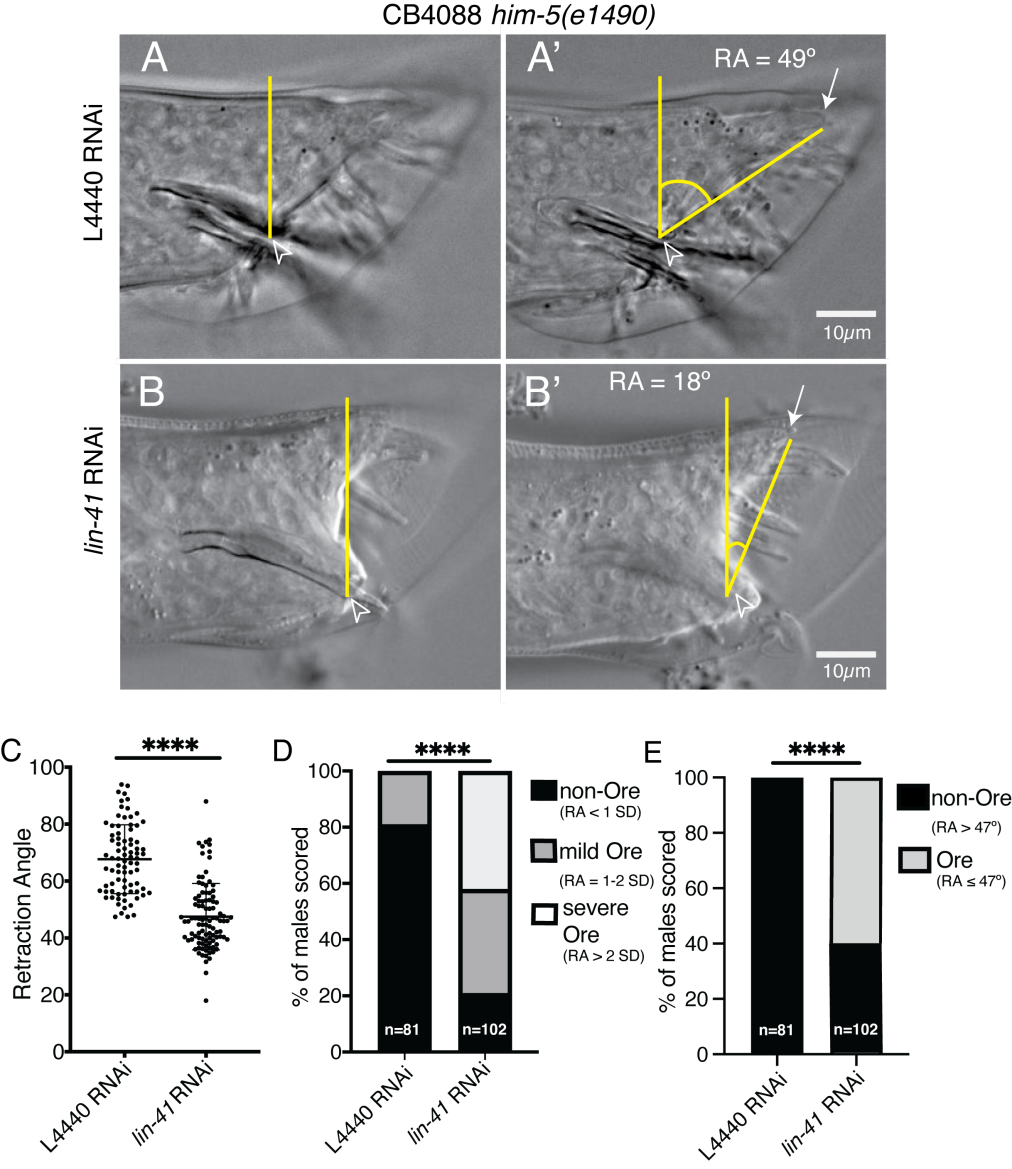
Newly hatched CB4088 *him-5(e1490)* males were fed bacteria carrying either the L4440 control plasmid or a *lin-41* RNAi-inducing plasmid and imaged as early adults. (A, B) Arrowhead indicates location of cloacal opening. Vertical yellow line is the cloacal axis, perpendicular to the anterior-posterior axis of the worm and originating at the cloacal opening. (A’, B’) Arrow indicates the posterior position of the male tail tip. Lines connect the male tail tip to the cloacal axis at the cloacal opening. The curve represents the Retraction Angle (RA). (C) The mean Retraction Angle among *lin-41* RNAi males is significantly lower than that of the L4440 control. Each dot represents an individual male worm. Graph shows mean and standard deviation. (Student’s t-test, **** p<0.0001). (D) Penetrance and severity of retraction phenotypes defined with respect to the mean RA of L4440 RNAi males where non-Ore male tails had an RA <1 standard deviation from the mean of the L4440 RNAi population, mild Ore had an RA between 1-2 standard deviations from the L4440 mean, and severe Ore had an RA >2 standard deviations from the L4440 mean (Fisher exact test, Ore and non-Ore, **** p<0.0001). (E) Penetrance of the Ore phenotype, defined as an RA less than the lowest RA in the L4440 population (47º) (Fisher exact test, Ore and non-Ore, **** p<0.0001).

## Description

During the final larval stage, the *C. elegans* male tail tip undergoes a dramatic morphological change, as four epidermal tail tip cells fuse and retract anteriorly to form a stereotypic rounded, peloderan tail (Nguyen et al., 1999) . This shape change is regulated by the heterochronic pathway, which coordinates stage-specific cell divisions, cell fate decisions, and morphogenetic movements during development (Del Rio-Albrechtsen et al., 2006) . Loss of the heterochronic gene, *lin-41* , causes precocious tail tip cell fusion and retraction in the early L3, resulting in an Over-retraction (Ore) phenotype characterized by a severely truncated male tail (Del Rio-Albrechtsen et al., 2006) . To date, the Ore phenotype has been described qualitatively. Here, we describe a method for quantitatively assessing the Ore phenotype by measuring the degree of tail tip retraction.

We hypothesized that measuring the position of the male tail tip relative to a structure that develops independently of the heterochronic pathway, the cloacal opening, would allow us to compare the degree of tail tip retraction between individuals and experimental conditions. For each male scored, we determined the angle between two lines, both originating at the cloacal opening. One line runs perpendicular to the anterior-posterior axis of the worm (“cloacal axis”) and the other runs to the posterior position of the tail tip. We call this measurement the “Retraction Angle,” because it indicates the extent to which the posterior edge of the fused tail tip cells is positioned relative to the position of the cloacal opening ( Figure 1A ). Because the tail tip over-retracts when LIN-41 function is compromised, (Del Rio-Albrechtsen et al., 2006) , we expected that the average Retraction Angle would be smaller in *lin-41* loss-of-function and RNAi conditions previously described as displaying the Ore phenotype compared to controls with wild-type levels of *lin-41* activity.

To test this hypothesis, we depleted *lin-41* by RNAi feeding in CB4088 *him-5(e1490) V* males and measured the Retraction Angle. As predicted, the average Retraction Angle was significantly lower in the *lin-41* RNAi treated cohort (47.37º +/- 11.57º; n=102) compared to the L4440 control (67.67º +/-12.12º; n=81). This suggests that, on average, the tail tips in males exposed to *lin-41* RNAi retracted more than those raised on control RNAi, consistent with the *lin-41* reduction-of-function precocious retraction phenotype ( Figure 1B,C ).

While the average Retraction Angle facilitates comparisons across populations, to calculate the penetrance of the Ore phenotype, individual male tails must be classified. Given the range of Retraction Angle observed in control RNAi conditions ( Figure 1C ), we consider several alternative approaches for establishing cutoff values based on the Retraction Angle in the control. In the first approach, any *lin-41* RNAi treated male with a Retraction Angle more than one standard deviation below the control Retraction Angle mean (55º) could be classified as Ore. According to this definition, 79% of males fed *lin-41* RNAi displayed the Ore phenotype ( Figure 1D ). This penetrance is similar to the previous report using qualitative scoring methods that 74% of CB4088 males treated with *lin-41* RNAi exhibit the Ore phenotype (Del Rio-Albrechtsen et al., 2006) . However, we note that variability within the L4440 control population accounts for the 19% that would be classified as Ore by this criterion. A more stringent criterion of 2 standard deviations reduces to zero the number of L4440 control males classified as Ore and provides a penetrance of 42% Ore in the *lin-41* RNAi condition. While this strict cutoff ensures that no control animals are classified as Ore, it may underestimate the true penetrance of the Ore phenotype in the *lin-41* RNAi condition. One way to capture the variability of the Ore phenotype in viewing the measurements would be to define a “severe” Ore phenotype as diverging from control by more than 2 standard deviations, and a “mild” Ore as diverging by 1-2 standard deviations from the control ( Figure 1D ). Another simpler approach is to set the smallest Retraction Angle in the L4440 control population as the threshold for Ore classification. Using this methodology, any animal with a Retraction Angle lower than 47º, 60% of the *lin-41* RNAi population in this case, would be classified as Ore ( Figure 1E ).

Here, we have shown that quantifying the Ore phenotype based on the degree of male tail tip retraction corresponds with published qualitative characterizations of the penetrance of the precocious morphology in RNAi experiments. Uncovering non-canonical roles for heterochronic pathway genes like *lin-41* is an ongoing research pursuit in the *C. elegans* community. In contrast to other heterochronic phenotypes that are difficult to assess like precocious alae, the Ore phenotype in the male tail tip is well-characterized, highly penetrant, and quantifiable. Therefore, our quantitative method for scoring the Ore phenotype is a convenient way to validate the efficacy of *lin-41* RNAi to aid in interpretation of experiments seeking to elucidate additional roles for this gene or for functionally related genes. In addition to reducing bias in scoring the Ore phenotype, our quantification of tail tip retraction also reveals variation in wild-type male tail morphogenesis. For those interested in male tail morphogenesis, our quantitative method for assessing the degree of male tail tip retraction may also be useful to characterize differences between strains or species.

## Methods

RNAi experiments were conducted as described by Timmons et al., 2001. A cohort of CB4088 *him-5(e1490)* *V* animals was obtained by subjecting gravid CB4088 hermaphrodites to hypochlorite treatment and incubating their embryos in M9 overnight at 20ºC. Synchronized L1s were transferred to standard RNAi plates seeded with HT115 carrying the empty vector L4440 plasmid (negative control), the *skn-1* plasmid (positive control), or the *lin-41* plasmid. Complete penetrance of the *skn-1* maternal lethal phenotype of dead embryos (Emb) confirmed the efficacy of our RNAi plates in each experimental replicate.

After 56-60 hours at 20ºC, young adult males fed L4440 and *lin-41* RNAi were transferred to 4% Agar pads, paralyzed in 10mM Levamisole (Sigma 5086-74-8), and z-stacks were collected on the Imager.Z1 compound microscope at 400x. All image analysis was performed on ImageJ, only on animals lying on their right or left side (79% of 102 control L4440 males and 92% of 138 *lin-41* RNAi males). Animals that were not lying on their side (that is, were viewed from the dorsal or ventral side) were excluded because this position obscures either the cloaca or the tail tip. Males that had arrested in the L4 or exploded through the cloaca, both severe *lin-41* reduction-of-function phenotypes, were also excluded from the analysis (Del Rio-Albrechtsen et al., 2006) . This exclusion represented 29% (n=138) of *lin-41* RNAi males examined, leaving 102 scoreable worms for the *lin-41* RNAi *.* Virtually all (98%, n=138) *lin-41* RNAi worms did not fully shed their cuticle, but this did not interfere with the measurements.

The Retraction Angle was quantified using the Angle Tool in ImageJ. Through each eligible image, two intersecting lines were drawn. In a z-slice where the cloacal opening was in focus, a line was drawn perpendicular to the anterior-posterior axis of the worm, with the origin at the cloacal opening. In a z-slice where the male tail tip was in focus, a second line was drawn connecting the male tail tip to the first line drawn, intersecting at the cloacal opening. The angle between these two lines was measured. All statistical analysis was performed on Graphpad Prism. The statistical significance of the numeric Retraction Angle data was derived from an Unpaired Student’s T-test with Welch’s Correction.

## Reagents

· CB4088 *him-5 ( e1490 )* *V*

· L4440 RNAi pPD129.36 (Timmons and Fire, 1998)

· *lin-41* RNAi AH sjj_C12C8.3 (Kamath et al., 2003)

· *skn-1* RNAi mv_T19E7.2 (Rual et al., 2004)

· Luria Broth + Ampicillin (50µg/mL)/Tetracycline(10µg/mL) agar plates

· LB + Ampicillin (100µg/mL) liquid

· Primers used to sequence *lin-41* RNAi reagent

o Left Oligo: sjj_C12C8.3_f: CCAAGTCCAAATGAGCCAAT

o Right Oligo: sjj_C12C8.3_b: ACTCAACCAACATCCCAAGC
